# Association of Prostate-Specific Antigen Velocity With Clinical Progression Among African American and Non-Hispanic White Men Treated for Low-Risk Prostate Cancer With Active Surveillance

**DOI:** 10.1001/jamanetworkopen.2021.9452

**Published:** 2021-05-17

**Authors:** Tyler J. Nelson, Juan Javier-DesLoges, Rishi Deka, P. Travis Courtney, Vinit Nalawade, Loren Mell, James Murphy, J. Kellogg Parsons, Brent S. Rose

**Affiliations:** 1 Department of Medicine, Veterans Health Administration San Diego Health Care System, La Jolla, California; 2 Department of Radiation Medicine and Applied Science, University of California, San Diego, School of Medicine, La Jolla; 3 Department of Urology, University of California, San Diego, School of Medicine, La Jolla

## Abstract

**Importance:**

The association of prostate-specific antigen velocity (PSAV) with clinical progression in patients with localized prostate cancer managed with active surveillance remains unclear and, to our knowledge, has not been studied in African American patients.

**Objectives:**

To test the hypothesis that PSAV is associated with clinical progression in patients with low-risk prostate cancer treated with active surveillance and to identify differences between African American and non-Hispanic White patients.

**Design, Setting, and Participants:**

This was a retrospective population-based cohort study using patient records from the Veterans Heath Administration Informatics and Computing Infrastructure on 5296 patients with a diagnosis of localized prostate cancer from January 1, 2001, to December 31, 2015, who were managed with active surveillance. Follow-up extended through March 31, 2020. Low-risk prostate cancer was defined as International Society of Urologic Pathology grade group (GG) 1 clinical tumor stage 2A or lower, PSA level of 10 ng/dL or lower, active surveillance, and no definitive treatment within the first year after diagnosis with at least 1 additional staging biopsy after diagnostic biopsy.

**Exposures:**

Prostate-specific antigen testing.

**Main Outcomes and Measures:**

The primary outcome was GG progression detected after repeated biopsy or prostatectomy, defined as GG2 or higher or GG3 or higher. The secondary outcome was incident metastases. Cumulative incidence functions and multivariable Cox proportional hazards regression models were used to test associations between PSAV and outcomes.

**Results:**

The final cohort (n = 5296) included 3919 non-Hispanic White men (74.0%; mean [SD] age, 65.7 [5.8] years) and 1377 African American men (26.0%; mean [SD] age, 62.8 [6.6] years). Compared with African American patients, non-Hispanic White patients were older (mean [SD] age, 65.7 [5.8] years vs 62.8 [6.6] years; *P* < .001), presented with higher cT stage (stage T2, 608 [15.5%] vs 111 [8.1%]; *P* < .001), had a higher Charlson Comorbidity Index score (1 and ≥2, 912 [23.3%] vs 273 [19.8%]; *P* = .002), had higher median income ($60 000 to ≥$100 000, 1223 [31.2%] vs 282 [20.5%]; *P* < .001), and had a higher median level of education (20% to ≥30% with college degree, 1192 [30.4%] vs 333 [24.2%]; *P* < .001). Progression to GG2 or higher occurred in 2062 patients (38.9%), with a cumulative incidence of 43.2%, and progression to GG3 or higher occurred in 728 patients (13.7%). Fifty-four patients (1.0%) developed metastases. On multivariable analysis, PSAV was significantly associated with progression to GG2 (hazard ratio, 1.32 [95% CI, 1.26-1.39]), GG3 (hazard ratio, 1.51 [95% CI, 1.41-1.62]), and metastases (hazard ratio, 1.38 [95% CI, 1.10-1.74]). Optimal PSAV thresholds that were associated with progression were significantly lower for African American patients (0.44 ng/mL/y) compared with non-Hispanic White patients (1.18 ng/mL/y).

**Conclusions and Relevance:**

This study suggests that PSAV is significantly associated with grade progression among patients with low-risk prostate cancer managed with active surveillance, but at lower values for African American patients compared with non-Hispanic White patients. These data suggest that serial PSA measures may potentially substitute for multiple prostate biopsies and that African American patients may merit increased frequency of PSA testing.

## Key Points

**Question:**

Is prostate-specific antigen velocity associated with clinical progression in patients with low-risk prostate cancer treated with active surveillance, and are there differences between African American and non-Hispanic White patients?

**Findings:**

In this population-based cohort study of 5296 patients with low-risk prostate cancer, prostate-specific antigen velocity was associated with clinical progression to more advanced disease. Compared with non-Hispanic White patients, African American patients were more likely to experience disease progression at lower prostate-specific antigen velocity thresholds.

**Meaning:**

This study suggests that prostate-specific antigen velocity is a useful clinical tool for all patients treated with active surveillance, and African Americans may require closer attention to follow-up.

## Teaser

This cohort study evaluates whether prostate-specific antigen velocity is associated with clinical progression in patients with low-risk prostate cancer treated with active surveillance and identifies differences between African American and non-Hispanic White patients.

## Introduction

Active surveillance (AS) is a preferred treatment strategy for appropriate patients with low-risk prostate cancer.^1,2,3,4,5^ Although surveillance protocols vary, prostate-specific antigen (PSA) testing and a periodic repeated staging biopsy are the 2 primary methods for detecting disease progression.^6^ Biopsies are effective for definitively demonstrating disease progression through pathologic upgrading, yet they are invasive and associated with a substantial risk of infection.^1^

Prostate-specific antigen testing is noninvasive, low risk, and readily obtained. However, the clinical utility of PSA—in particular, PSA velocity (PSAV)—and its association with disease progression remain a focus of debate.^1,2,3,4,5^ Prostate-specific antigen velocity is the rate of PSA change over time. Although some studies have demonstrated prognostic benefit for PSAV with clinical progression, others have not. Moreover, to our knowledge, there are no published PSAV data for African American patients undergoing AS. Studies have indicated that, compared with non-Hispanic White patients, African American patients undergoing AS have comparable disease survival but are more likely to clinically progress.^6,7,8,9^ Further study of PSAV may potentially inform the care of African American patients through improved AS protocols.

The aim of this study was to evaluate whether PSAV is associated with pathologic progression among African American and non-Hispanic White patients with low-risk prostate cancer managed with AS. The hypothesis was that PSAV would be associated with pathologic progression independent of race.

## Methods

### Data Collection and Study Population

We performed an analysis using the Veterans Health Administration (VHA) Corporate Data Warehouse (CDW) and accessed data through the VHA Informatics and Computing Infrastructure.^10^ The CDW contains electronic health records of more than 9 million veterans who received care at approximately 1255 health care facilities, including 170 medical centers and 1074 outpatient clinics throughout the United States. This study was reviewed and approved by the VHA San Diego Health Care System.^11^ Waivers of consent and authorization were granted by the institutional review board and the Research and Development Committee of the VHA San Diego Health Care System because access to the data was restricted to those with VHA certifications and behind the VHA firewall. This study followed the Strengthening the Reporting of Observational Studies in Epidemiology (STROBE) reporting guideline for cohort studies.

African American and non-Hispanic White patients in the VHA with a diagnosis of pathologically confirmed low-risk prostate cancer who were managed with AS for at least 1 year were identified between January 1, 2001, and December 31, 2015. Low-risk prostate cancer was defined as International Society of Urologic Pathology grade group (GG) 1 (Gleason score of 6), clinical tumor stage 2A or lower, and PSA level of 10 ng/dL or lower (to convert to micrograms per liter, multiply by 1.0).^12^ Active surveillance was defined as no definitive treatment within the first year after diagnosis and at least 1 additional staging biopsy after diagnostic biopsy. Inclusion in the analytic cohort required that a patient have at least 3 separate PSA values collected over a minimum of 12 months, 1 of which was obtained prior to prostate cancer diagnosis. Patients with previous pelvic radiotherapy or missing demographic covariates and patients who were neither non-Hispanic White nor African American were excluded. Follow-up occurred from January 1, 2001, to March 31, 2020.

### Primary Outcome: Upgrading

The primary outcome was incident disease progression on repeated biopsy or radical prostatectomy, whichever occurred first. If a patient underwent prostatectomy having not previously been upgraded by staging biopsy, and the prostatectomy specimen demonstrated upgrading, then time of upgrading was defined as time of prostatectomy. Patients who received definitive treatment before pathologic progression were censored at the time of definitive treatment. Pathologic GG was obtained through the use of natural language processing on all biopsy and prostatectomy reports. To confirm the validity of the natural language processing, 100 patients were randomly selected from the cohort of patients with low-risk prostate cancer, and GG scores were concordant in 95% of cases, with no differences in accuracy between African American and non-Hispanic White patients.

Definitive treatment was identified through analysis of diagnosis and procedural codes and then augmented by manual review. *Current Procedural Terminology*; *International Classification of Diseases, Ninth Revision, Clinical Modification* (*ICD-9*); and *International Statistical Classification of Diseases and Related Health Problems, Tenth Revision* (*ICD-10*) diagnosis and procedure codes were first searched from outpatient and inpatient data in the VHA CDW to assess receipt of radiotherapy or radical prostatectomy. To identify definitive treatment received outside the VHA, the same diagnosis and procedure codes were searched in the outpatient and inpatient files in the Centers for Medicare & Medicaid Services database. Finally, records of patients with a decrease in PSA level of at least 50% from diagnosis were manually reviewed at any time after prostate cancer diagnosis.

### Secondary Outcome: Metastases

The secondary outcome was incident metastatic disease. The electronic medical records of all patients meeting any of the following criteria were manually reviewed: (1) *ICD-9* or *ICD-10* codes for metastasis of the bone or malignant neoplasm of nonpelvic lymph nodes, (2) PSA level higher than 20 ng/dL, and (3) receipt of androgen deprivation therapy in the form of a gonadotropin-releasing hormone agonist or antagonist. Records of patients with a PSA level higher than 20 ng/dL were manually reviewed owing to classification of these patients as being at high risk of progression per National Comprehensive Cancer Network guidelines.^12^ An additional 100 medical records that did not fulfill these criteria were reviewed, and no cases of metastases were identified.

Potential confounding variables, such as smoking, statin use, and alcohol use, were found through a search of *ICD-9* and *ICD-10* codes and medication data through the VHA CDW. Data on statin use and Charlson Comorbidity Index were obtained for addition into multivariable models to account for competing mortality and confounding. Income and educational level data were based on zip code–level data and were stratified above or below the median for the cohort.

### PSAV Calculations

Prostate-specific antigen measures were included in a linear regression model to calculate each patient’s PSAV. The baseline PSA was defined as the last PSA measure prior to diagnosis of low-risk prostate cancer. The last PSA measure was defined as the last PSA measure before the upgrading event, treatment, administration of 5-alpha reductase inhibitors (5-ARIs), or transurethral resection of the prostate. We defined PSAV as the change in PSA level (nanograms per deciliter) per year, as measured by the slope of the linear regression, using a minimum of 3 measures over a minimum of 1 year.^13^ Prostate-specific antigen values recorded after disease progression detected on biopsy, definitive treatment with surgery or radiotherapy, incident metastatic disease, administration of 5-ARIs, or transurethral resection of the prostate were excluded from analysis. To maximize clinical utility in this population, we chose to use absolute change in PSA without log transformation or calculating percentage change.

### Statistical Analysis

Cumulative incidence functions and multivariable Cox proportional hazards regression models were used to test associations between PSAV and outcomes. Mortality from other causes was censored. Time to upgrading event was defined as time from initial diagnosis until date of biopsy or prostatectomy upgrading. Patients without any upgrading events were censored at last follow-up or receipt of definitive treatment. Time to metastasis event was defined as time from initial diagnosis until diagnosis of metastases. Patients without any events were censored at last follow-up.

Multivariable Cox proportional hazards regression models included age, race, baseline PSA level, last PSA level, modified Charlson Comorbidity Index score (0, 1, and ≥2), clinical T stage, statin use, median income, median educational level, and the interaction term African American race × PSAV, based on prior publications.^14^ These variables were selected by backward model selection. Patients who were given 5-ARIs or underwent transurethral resection of the prostate were still included, but subsequent PSAs were censored after surgery or medication initiation. The last PSA measure was included in multivariable models to directly compare the strength of associations between PSAV and PSA level with outcomes.

Cumulative incidence of GG2 and GG3 upgrading at 7 years stratified by optimal PSAV threshold was selected to be the primary end point. Owing to the observation of a significant interaction term between race and PSAV in multivariable Cox proportional hazards regression models, optimal PSAV thresholds were assessed separately for African American patients and non-Hispanic White patients. Optimal PSAV thresholds were assessed based on the greatest separation of the cohorts using maximally selected rank statistics. Maximally selected rank statistics allow for the evaluation of thresholds and stratify continuous variables into 2 groups based on a threshold that will keep the groups furthest apart.^15^ All statistical analyses were performed using R, version 3.6.1 (R Group for Statistical Computing). All *P* values were from 2-sided tests, and results were deemed statistically significant at *P* < .05.

## Results

### Study Population

**Figure 1.**
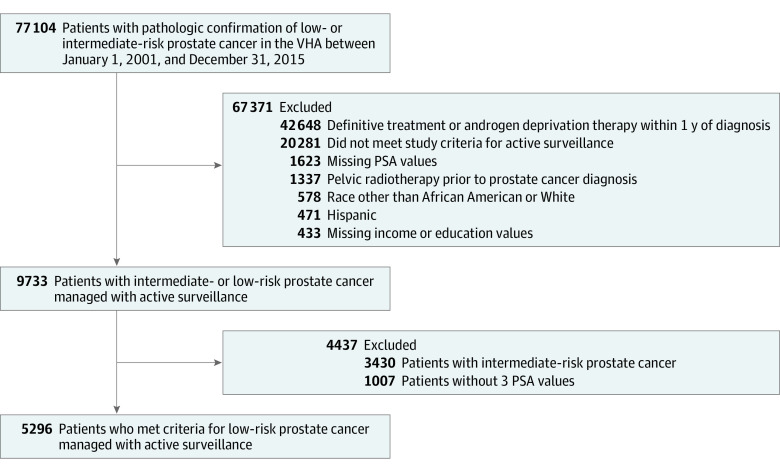
CONSORT Flow Diagram PSA indicates prostate-specific antigen; VHA, Veterans Health Administration.

The final cohort for baseline characteristics included 5296 patients: 3919 non-Hispanic White men (74.0%; mean [SD] age, 65.7 [5.8] years) and 1377 African American men (26.0%; mean [SD] age, 62.8 [6.6] years) (Figure 1 and Table 1). The mean (SD) follow-up was 7.91 (2.95) years. The mean (SD) number of PSA values used for PSAV calculations per patient was approximately 8.67 (5.16).

**Table 1.** Comparison of Demographic and Clinical Characteristics by Race

Baseline characteristic	Patients, No. (%)			*P* value
	Total (N = 5296)	Non-Hispanic White (n = 3919)	African American (n = 1377)	
Age, mean (SD), y	64.9 (6.1)	65.7 (5.8)	62.8 (6.6)	<.001
CCI score				
0	4111 (77.6)	3007 (76.7)	1104 (80.2)	.002
1	996 (18.8)	779 (19.9)	217 (15.8)	
≥2	189 (3.6)	133 (3.4)	56 (4.1)	
Median income, $				
<30 000	377 (7.1)	142 (3.6)	235 (17.1)	<.001
30 000 to <60 000	3414 (64.5)	2554 (65.2)	860 (62.5)	
60 000 to <100 000	1360 (25.7)	1099 (28.0)	261 (19.0)	
≥100 000	145 (2.7)	124 (3.2)	21 (1.5)	
Median education by % with college degree				
<10%	1170 (22.1)	776 (19.8)	394 (28.6)	<.001
10% to <20%	2601 (49.1)	1951 (49.8)	650 (47.2)	
20% to <30%	1170 (22.1)	912 (23.3)	258 (18.7)	
≥30%	355 (6.7)	280 (7.1)	75 (5.4)	
Statin use				
Yes	2774 (52.4)	2084 (53.2)	690 (50.1)	.05
No	2522 (47.6)	1835 (46.8)	687 (49.9)	
Year of diagnosis				
2001-2005	373 (7.0)	298 (7.6)	75 (5.4)	<.001
2006-2010	1759 (33.2)	1344 (34.3)	415 (30.1)	
2011-2015	3164 (59.7)	2277 (58.1)	887 (64.4)	
Clinical T stage at diagnosis				
1	4577 (87.4)	3311 (85.5)	1266 (91.9)	<.001
2	719 (13.6)	608 (15.5)	111 (8.1)	<.001
Baseline PSA level, mean (SD), ng/dL	5.40 (1.90)	5.38 (1.91)	5.44 (1.88)	.37
Last PSA level, mean (SD), ng/dL	7.59 (5.55)	7.33 (5.37)	8.35 (5.98)	<.001
No. of PSA values included in PSAV calculation, mean (SD)	8.67 (5.16)	8.93 (5.36)	7.91 (4.47)	<.001
Total follow-up time, mean (SD), y	7.91 (2.95)	7.96 (3.01)	7.76 (2.75)	.03
Time to upgrading event, mean (SD), y	4.46 (2.76)	4.50 (2.81)	4.35 (2.61)	.10
PSA velocity, mean (SD)^a^	0.62 (1.34)	0.53 (1.27)	0.87 (1.48)	<.001
Definitive treatment				
Yes	2230 (42.1)	1533 (39.1)	697 (50.6)	<.001
No	3066 (57.9)	2386 (60.9)	680 (49.4)	

Abbreviations: CCI, Charlson Comorbidity Index; PSA, prostate-specific antigen; PSAV, prostate-specific antigen velocity.

SI conversion factor: To convert PSA level to micrograms per liter, multiply by 1.0.

^a^ Defined as the change in PSA level (ng/dL) per year.

Compared with African American patients, non-Hispanic White patients were older (mean [SD] age, 65.7 [5.8] years vs 62.8 [6.6] years; *P* < .001), presented with higher cT stage (stage T2, 608 [15.5%] vs 111 [8.1%]; *P* < .001), had a higher Charlson Comorbidity Index score (1 and ≥2, 912 [23.3%] vs 273 [19.8%]; *P* = .002), had higher median income ($60 000 to ≥$100 000, 1223 [31.2%] vs 282 [20.5%]; *P* < .001), and had a higher median level of education (20% to ≥30% with college degree, 1192 [30.4%] vs 333 [24.2%]; *P* < .001) (Table 1). The last PSA level was significantly lower prior to biopsy or prostatectomy in non-Hispanic White patients than in African American patients (mean [SD], 7.33 [5.37] ng/dL vs 8.35 [5.98] ng/dL; *P* < .001).

### Primary Outcome: Upgrading

Progression to GG2 or higher occurred in 2062 patients (38.9%): 1980 patients (96.0%) at the time of restaging biopsy and 82 patients (4.0%) at the time of prostatectomy. The cumulative 7-year incidence of progression to GG2 or higher was 43.2%. Progression to GG3 or higher occurred in 728 patients (13.7%): 700 patients (96.2%) at restaging biopsy and 28 patients (3.8%) at prostatectomy.

In unadjusted analyses, African American patients were significantly more likely than non-Hispanic White patients to progress to GG2 (cumulative percentages, 53% vs 40%; *P* < .001) and GG3 (cumulative percentages, 22% vs 17%; *P* < .001) disease. In multivariable regression, higher PSAV was associated with increased risks of progression to GG2 (hazard ratio [HR], 1.32; 95% CI, 1.26-1.39; *P* < .001) and GG3 (HR, 1.51; 95% CI, 1.41-1.62; *P* < .001) (Table 2). After controlling for the PSAV, we found that a higher last absolute PSA level was associated with a decreased risk of progression to GG2 (HR, 0.98; 95% CI, 0.97-0.99; *P* < .001) and GG3 (HR, 0.98; 95% CI, 0.97-0.99; *P* = .002), which suggests that a high but stable PSA level is not strongly associated with grade progression.

**Table 2.** Cox Proportional Hazards Regression Multivariable Model for Upgrading and Development of Metastatic Disease

Covariate	Grade group 2 upgrading		Grade group 3 upgrading		Metastasis	
	HR (95% CI)	*P* value	HR (95% CI)	*P* value	HR (95% CI)	*P* value
Age, increasing	0.99 (0.99-1.00)	.06	1.01 (0.99-1.02)	.13	1.03 (0.99-1.07)	.21
CCI score						
1 vs 0	1.03 (0.92-1.16)	.59	1.01 (0.84-1.22)	.91	0.79 (0.36-1.72)	.56
≥2 vs 0	1.03 (0.81-1.32)	.78	1.28 (0.88-1.86)	.20	4.69 (2.17-10.13)	.02
Median income, low vs high^a^	1.07 (0.98-1.16)	.14	0.95 (0.77-1.13)	.59	0.93 (0.23-1.63)	.85
Median educational level, low vs high^b^	0.82 (0.71-0.93)	.01	0.84 (0.79-1.03)	.09	1.16 (0.06-2.26)	.60
Statin use, yes vs no	0.99 (0.91-1.09)	.94	1.02 (0.88-1.19)	.78	1.11 (0.64-1.91)	.71
Clinical stage, T2 vs T1	0.87 (0.7-0.99)	.05	0.96 (0.78-1.19)	.73	1.69 (0.90-3.19)	.10
Last PSA level, increasing	0.98 (0.97-0.99)	<.001	0.98 (0.97-0.99)	.002	1.01 (0.98-1.05)	.46
PSAV, increasing^c^	1.32 (1.26-1.39)	<.001	1.51 (1.41-1.62)	<.001	1.38 (1.10-1.74)	.005
African American vs non-Hispanic White	1.42 (1.26-1.59)	<.001	1.51 (1.24-1.85)	<.001	0.83 (0.35-1.95)	.67
PSAV × African American race	0.92 (0.87-0.98)	.007	0.86 (0.78-0.94)	.001	1.02 (0.74-1.39)	.90

Abbreviations: CCI, Charlson Comorbidity Index; HR, hazard ratio; PSA, prostate-specific antigen; PSAV, prostate-specific antigen velocity.

^a^ Income was assessed by dividing the cohort into 2 equal-sized groups based on median income by zip code.

^b^ Educational level was assessed by dividing the cohort into 2 equal-sized groups based on median college graduation rate by zip code.

^c^ Defined as the change in PSA level (ng/dL [to convert to micrograms per liter, multiply by 1.0]) per year.

After comparing African American patients with non-Hispanic White patients, we found that African American patients had a higher risk of progression to GG2 (HR, 1.42; 95% CI, 1.26-1.59) and GG3 (HR, 1.51; 95% CI, 1.24-1.85). The interaction term between PSAV and African American race was significant for GG2 (HR, 0.92; 95% CI, 0.87-0.98; *P* = .009) and GG3 (HR, 0.86; 95% CI, 0.78-0.94; *P* = .002), indicating the need for different PSAV thresholds for African American and non-Hispanic White patients. Using maximally selected rank statistics, we found that the optimal PSAV threshold for GG3 upgrading was 0.44 ng/mL/y for African American patients and 1.18 ng/mL/y for non-Hispanic White patients.

**Figure 2.**
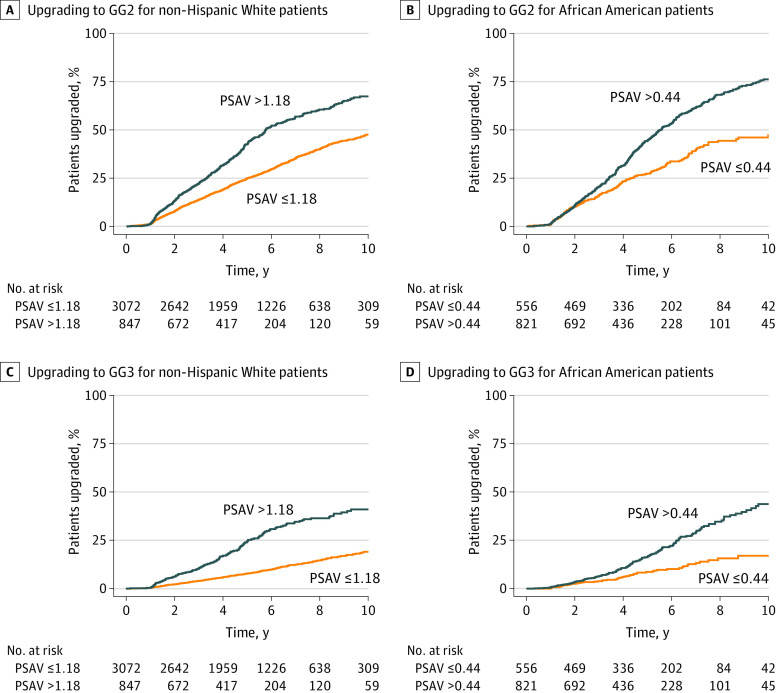
Cumulative Probability of Upgrading A, Upgrading to grade group (GG) 2 for non-Hispanic White patients. B, Upgrading to GG2 for African American patients. C, Upgrading to GG3 for non-Hispanic White patients. D, Upgrading to GG3 for African American patients. A prostate-specific antigen velocity (PSAV) of 1.18 or less for non-Hispanic White patients or 0.44 or less for African American patients is below the PSAV. A PSAV of greater than 1.18 for non-Hispanic White patients or greater than 0.44 for African American patients is above the PSAV threshold.

Cumulative 7-year incidences of GG2 upgrading below and above the PSAV threshold of 0.44 ng/mL/y were 39.8% and 61.4% (*P* < .001), respectively, for African American patients. Cumulative 7-year incidences of GG2 upgrading below and above the PSAV theshold of 1.18 ng/mL/y were 32.8% and 54.5% (*P* < .001), respectively, for non-Hispanic White patients (Figure 2A and B). Among African American patients, cumulative 7-year incidences of GG3 upgrading below and above the optimal PSAV threshold were 13.3% and 29.1% (*P* < .001), respectively. Among non-Hispanic White patients, cumulative 7-year incidences of GG3 upgrading below and above the optimal PSAV threshold were 12.5% and 34.6% (*P* < .001), respectively (Figure 2C and D).

### Secondary Outcome: Metastases

**Figure 3.**
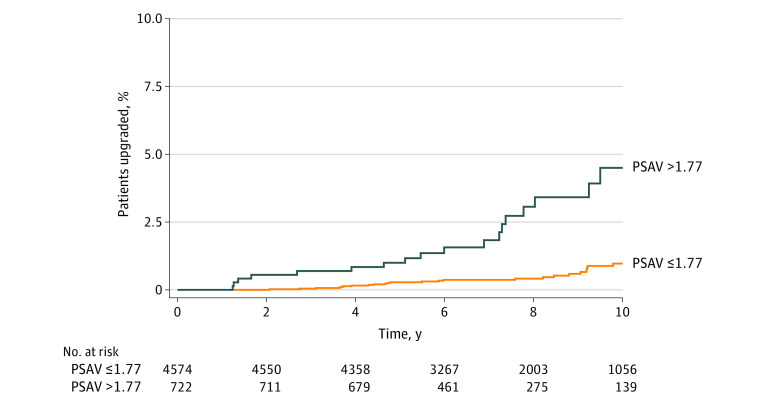
Cumulative Incidence of Metastases for All Patients Stratified by Prostate-Specific Antigen Velocity (PSAV) Threshold A PSAV of 1.77 or less is below the PSAV threshold. A PSAV of greater than 1.77 is above the PSAV threshold.

Metastases were observed in 54 patients (1.0%), with a cumulative 10-year incidence of 1.4%. In multivariable models, PSAV was associated with an overall 38% increased risk of metastases (HR, 1.38; 95% CI, 1.10-1.74; *P* = .005) (Table 2). There were no significant differences in the risks of metastases between African American patients and non-Hispanic White patients (HR, 0.83; 95% CI, 0.35-1.95; *P* = .67). The association between PSAV and metastases did not differ by race. The optimal PSAV threshold for metastases was found to be 1.77 ng/mL/y in the whole cohort (Figure 3). Cumulative incidences of metastases at 7 years were 0.4% and 1.8% (*P* < .001). At 10 years, cumulative incidences of metastases were 1.0% and 4.5% (*P* < .001).

## Discussion

In this racially diverse, national cohort of patients with low-risk prostate cancer undergoing AS, PSAV was associated with pathologic grade progression and risk of metastases. In multivariable models including both absolute PSA and PSAV values, PSAV was consistently more associated with increased progression to GG2, GG3, and metastases. Compared with non-Hispanic White patients, African American patients were more likely to progress to higher-grade disease at lower values of PSAV and had lower PSAV thresholds for upgrading.

To our knowledge, this study is the first to assess PSAV in African American patients undergoing AS, and one of the largest studies yet of PSAV, progression, and AS in patients with localized prostate cancer. These results suggest at least 2 ways in which PSAV may substantively inform the clinical care of these patients. First, given the robustness of PSAV’s association with pathologic upgrading, these data warrant consideration of substituting PSAV for serial prostate biopsies in appropriately selected patients. Prostate-specific antigen velocity is readily calculated from easily accessible online calculators and can be accessed in a clinical setting.^16^ Transrectal ultrasonography-guided prostate biopsies routinely performed for these patients are invasive and associated with significant risks of hospitalization for urinary sepsis with panresistant microbes,^12,17,18^ and methods for diminishing biopsy use would thus improve care. Second, African American patients may merit increased frequency of PSA testing, with use of correspondingly lower PSAV thresholds to inform clinical decision-making. Based on this study, a PSAV threshold of 0.44 ng/mL/y suggests a clinically significant risk of progression for African American patients compared with 1.18 ng/mL/y for non-Hispanic White patients. These findings are further supported by a recent publication demonstrating that AS is safe for African American patients, although they are at increased risk of progression.^19^ Further prospective studies will be needed to validate these thresholds.

Our study is unique in that all of the patients are members of a relatively equal-access health care system. Our findings add to the literature, which is inconsistent on whether African American patients are at increased risk for adverse outcomes during AS for low-risk prostate cancer.^5^ Prior studies have shown that the participation rate of African American patients in studies on AS is low.^8,19^ In a more recent study^9^ of a cohort of 355 African American patients (39.7%) and 540 White patients (60.3%), the authors found no differences in pathologic upgrading for patients undergoing AS, but they did not evaluate PSAV. Unlike in that study, we found differences in pathologic upgrading between African American patients and non-Hispanic White patients; however, we did not find differences in metastasis. Together, both studies demonstrate the importance of having well-balanced cohorts and that AS is safe for African American patients.

The role of using PSAV to inform decision-making in an AS program has remained unclear.^19,20^ The National Comprehensive Cancer Network and the American Urological Association evidence-based clinical guideline for the treatment of prostate cancer do not comment on the use of PSAV during AS.^12,21^ In contrast, the European Association of Urology recently released guidelines recommending PSA progression as an indication for biopsy for patients undergoing AS.^22^ Limitations of prior PSAV studies have confounded assessments of its clinical utility. These limitations include inconsistent criteria for identifying patients who should receive AS, homogeneous study populations overwhelmingly composed of non-Hispanic White patients, and variable definitions of PSAV,^23^ with at least 3 different methods of calculation reported.^13,24^

Another notable limitation of prior studies is the use of small numbers of PSA values to calculate PSAV. At least 3 prior studies have calculated PSAV using only 2 PSA values measured over a 12-month period.^13,25,26^ Because PSA values may vary by as much as 20% from one measure to the next, the use of limited ranges of PSA values in PSAV calculations will result in less precise PSAV estimates. Moreover, some prior studies did not report the number of PSA values used in PSAV calculations, underscoring the uncertainty of these measures and rendering problematic the interpretation of their clinical significance.^27,28^

### Strengths and Limitations

Our study calculated PSAV using a minimum of 3 PSA values collected over at least a 12-month period using the slope of the linear regression of PSA, which is considered a reference standard for PSAV calculation.^13^ In testing associations between PSAV and length of follow-up and number of PSA values used to calculate PSAV, we found no associations that may indicate bias in the ascertainment of PSAV. Other strengths of this analysis include strictly defined inclusion and exclusion criteria consistent with current community standards of care for AS; the most racially diverse PSAV study population to date, composed of more than 25% African American individuals in an equal-access health care system^5,12,25^; and inclusion of more than 10 years of cumulative incidence data for progression and incident metastases.

This study also has some limitations. One potential limitation is that the indications for which urologists performed follow-up biopsies could not be determined, which may have led to information bias. However, this bias would most likely have been nondifferential with respect to the primary and secondary outcomes. A second limitation is that it remains unclear as to how adjusted PSAV cut points may align with other prognostic variables for progression in AS or potentially inform clinical nomograms for progression. Third, it is possible that differential follow-up occurred between African American and non-Hispanic White patients, which may have led to ascertainment bias in assessment of the outcomes. Yet there were no clinically significant differences between African American and non-Hispanic White patients in duration of follow-up, mean PSA values, or time to upgrading event, suggesting that the intensity of follow-up did not differ by race (Table 1). Fourth, data on PSA density and percentage of positive biopsy results were not available, thus limiting this analysis. Fifth, we chose not to calculate PSAV as a doubling time or as a percentage change because they are difficult to assess in this cohort. In our cohort, the mean PSA level increased only from 5.40 to 7.59 ng/dL, which means that most patients would not ever have their PSA level double. Sixth, the number of metastatic events was low and therefore may not reach the number necessary to assess a difference between the 2 groups of patients, and this may be due to overspecification. Seventh, African American patients had different baseline demographic characteristics, which may have been associated with selection bias. Eighth, a potential limitation is that we did not have data triggering treatment other than biopsy results. However, there is no evidence that anxiety or other factors are associated with treatment decisions.^29^

## Conclusions

Prostate-specific antigen velocity was associated with pathologic grade progression among African American and non-Hispanic White patients with low-risk prostate cancer managed with AS, with grade progression occurring at lower PSAV values for African American patients. These data suggest that serial PSAV measures may potentially substitute for repeated prostate biopsies and that African American patients may merit increased frequency of PSA testing.
